# comoR: a software for disease comorbidity risk assessment

**DOI:** 10.1186/2043-9113-4-8

**Published:** 2014-05-23

**Authors:** Mohammad Ali Moni, Pietro Liò

**Affiliations:** 1 Computer Laboratory, University of Cambridge, William Gates Building, 15 JJ Thomson Avenue, Cambridge CB3 0FD, UK; 2 Department of Computer Science & Engineering, Pabna University of Science & Technology, Pabna, Bangladesh

**Keywords:** Comorbidities, Relative risk, Disease associations

## Abstract

**Background:**

The diagnosis of comorbidities, which refers to the coexistence of different acute and chronic diseases, is difficult due to the modern extreme specialisation of physicians. We envisage that a software dedicated to comorbidity diagnosis could result in an effective aid to the health practice.

**Results:**

We have developed an R software comoR to compute novel estimators of the disease comorbidity associations. Starting from an initial diagnosis, genetic and clinical data of a patient the software identifies the risk of disease comorbidity. Then it provides a pipeline with different causal inference packages (e.g. pcalg, qtlnet etc) to predict the causal relationship of diseases. It also provides a pipeline with network regression and survival analysis tools (e.g. Net-Cox, rbsurv etc) to predict more accurate survival probability of patients. The input of this software is the initial diagnosis for a patient and the output provides evidences of disease comorbidity mapping.

**Conclusions:**

The functions of the comoR offer flexibility for diagnostic applications to predict disease comorbidities, and can be easily integrated to high–throughput and clinical data analysis pipelines.

## Introduction

The term “comorbidity” refers to the coexistence or presence of multiple diseases or disorders in relation to a primary disease or disorder in a patient [1]. Multimorbidity can be also defined as coexistence of two or more diseases, but no index disease is considered [2]. A comorbidity relationship between two diseases exists whenever they appear simultaneously in a patient more than chance alone. It represents the co–occurrence of diseases or presence of different medical conditions one after another in the same patient [3,4]. Some diseases or infections can coexist in one person by coincidence, and there is no pathological association among them. However, in most of the cases, multiple diseases (acute or chronic events) occur together in a patient because of the associations among diseases. These associations can be due to direct or indirect causal relationships and the shared risk factors among diseases [5,6]. For an instance, people with HIV-1 appear to have a markedly higher rate of end-stage renal disease (ESRD) than the healthy people [7]. It is because some of the risk factors associated with HIV-1 acquisition are the same as those that lead to kidney disease. Patients with chronic kidney disease increase risk of cardiovascular mortality [8]. Thus HIV-1 infections is associated with cardiovascular mortality.

One of the most challenging problems in biomedical research is to understand the complex correlation mechanisms of human diseases. Recent research has increasingly demonstrated that many seemingly dissimilar diseases have common molecular mechanisms. Exploring relations between genes and diseases at the molecular level could greatly facilitate our understanding of pathogenesis, and eventually lead to better diagnosis and treatment. Diseases are more likely to be comorbid if they share associated genes [3]. However, some diseases have direct positive association among them while other diseases may have indirect positive association among them through the biological pathways. The analysis of pathway-disease associations, in addition to gene-disease associations, could be used to clarify the molecular mechanism of a disease. Ashley, Butte, Wheeler, Chen, Klein, Dewey, Dudley, Ormond, Pavlovic, Morgan, Pushkarev, Neff, Hudgins, Gong, Hodges, Berlin, Thorn, Sangkuhl, Hebert, Woon, Sagreiya, Whaley, Knowles, Chou, Thakuria, Rosenbaum, Zaranek, Church, Greely and Quake et al. analysed personal genome, gene-environment interactions and conditionally dependent risks for the clinical assessment [9]. Population-based disease association is also useful in conjunction with molecular and genetic data to discover the molecular origins of disease and disease comorbidity [4]. Patient medical records contain important clarification regarding the co-occurrences of diseases affecting the same patient. To estimate the correlation starting from disease co-occurrence, we need to quantify the strength of the comorbidity risk. Disease Ontology (DO) is also helpful to promote the investigation of diseases and disease risk factors [10].

Comorbidity is an important factor for better risk stratification of patients and treatment planning. The more precise predictions can be made by taking comorbidity into account, the more accurate patient management could be possible. Comorbidity has a significant predictive value on overall survival [11]. Older persons’ survival is highly dependent on it. Comorbidities influence patients treatments and confound survival analysis [12]. For an instance, comorbidity has a major effect on survival in gynaecological cancer, particularly for cancer of the cervix [13]. Many researchers have developed survival analysis software for predicting outcomes of the disease [14-23]. However, all of them are based on the single disease. But survival of patient depends on the disease comorbidity, environment, patient age and treatment plan. Kan et al. performed survival analysis of elderly dialysis patients considering comorbidity risk [24]. They observed that the life expectancy decreases with increasing the number of comorbid diseases. So it is important to consider the comorbidity for more accurate survival prediction.

We have developed an R software comoR to compute statistically significant associations among diseases and to predict disease comorbidity risk by using diverse set of data. The input of this software is the initial diagnosis for a patient. To perform the computation of the comorbidity risk, this software uses clinical, gene expression, pathways and ontology data. It provides different comorbidity assessment; integration of genetic information with the comoR output data could be used to infer causal relationships among diseases and to predict more accurate survival probability of patients. The goal of this software is to assist a medical practitioner in decision making in potential treatment.

## Implementation

**Figure 1:**
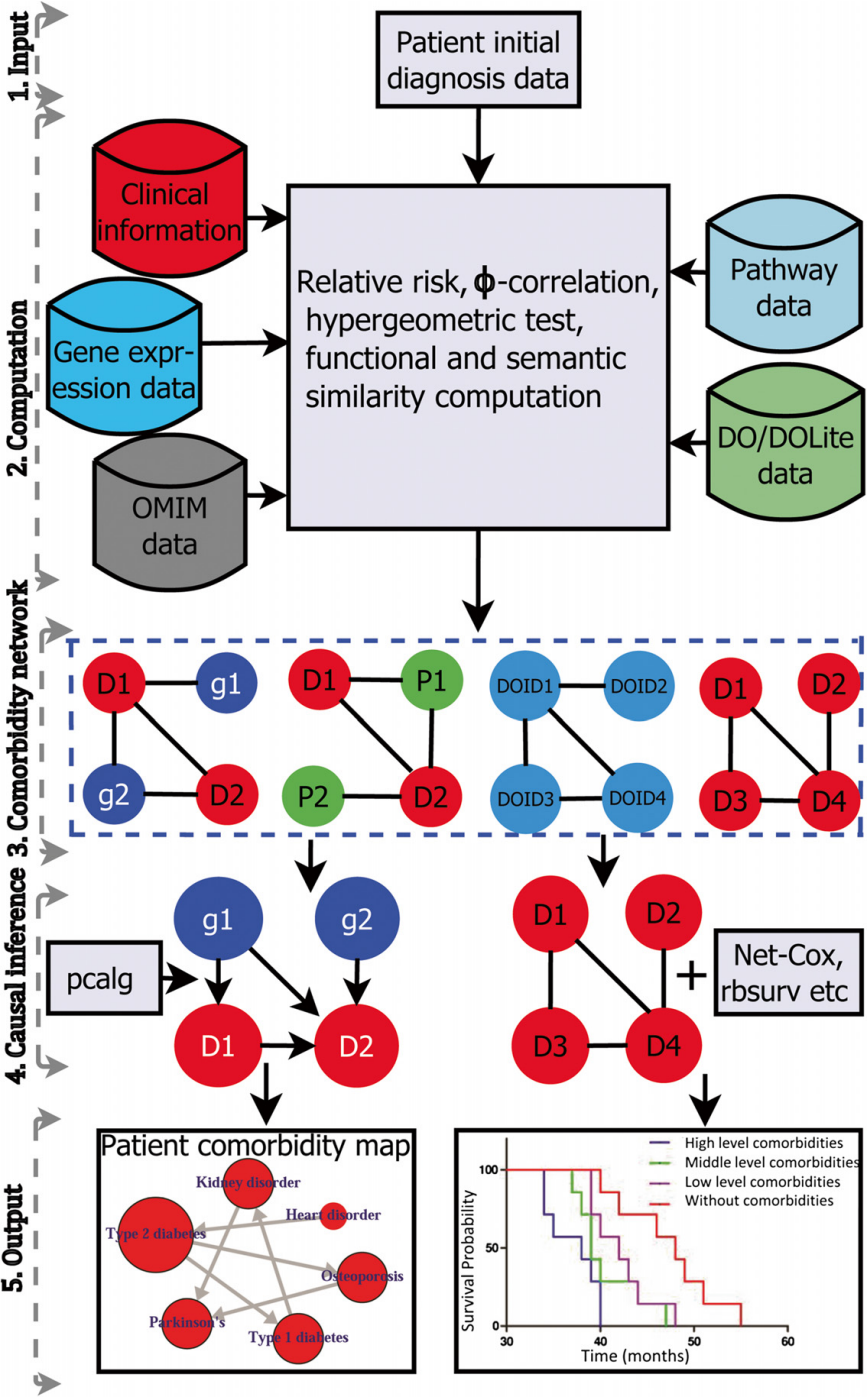
**Flow diagram of the **comoR**software.** Step 1: comoR takes as input preliminary diagnosis data of a patient. Step 2: It preprocesses and updates required databases, performs statistical computation (hypergeometric and semantic similarity tests), and calculates relative risks and *ϕ*-correlation (Pearsons correlation for binary variables) between diseases. Step 3: Comorbidity scores and disease network are provided as a result to the user. Step 4: Causal inference graphical models with the R package pcalg. Step 5: Visualisation of the comorbidity map and survival probability of patient considering comorbidity4. This map could be extended to incorporate diet and exercise as in [9]. Symbols D, g, P and DOID are used to indicate disease, gene, pathway and disease ontology id respectively.

The comoR provides a number of processing options to find comorbidity of a disease. R bioconductor annotation data packages “org.Hs.eg.db” and “DO.db” are used for the annotation and mapping between gene symbol, Entrez id, OMIM (Online Mendelian Inheritance in Man) id and DO (Disease Ontology) term [25]. comoR is also dependent on “DOSE” bioconductor package for the mapping of DO and DOLite [26]. A set of differential expressed gene symbols/Entrez ids/OMIM id/3 or 5 digit ICD-9-CM code of the disease can be used as input of comoR functions. Flow diagram of the comoR software is shown in Figure 1.

### Comorbidity based on clinical information

**Figure 2:**
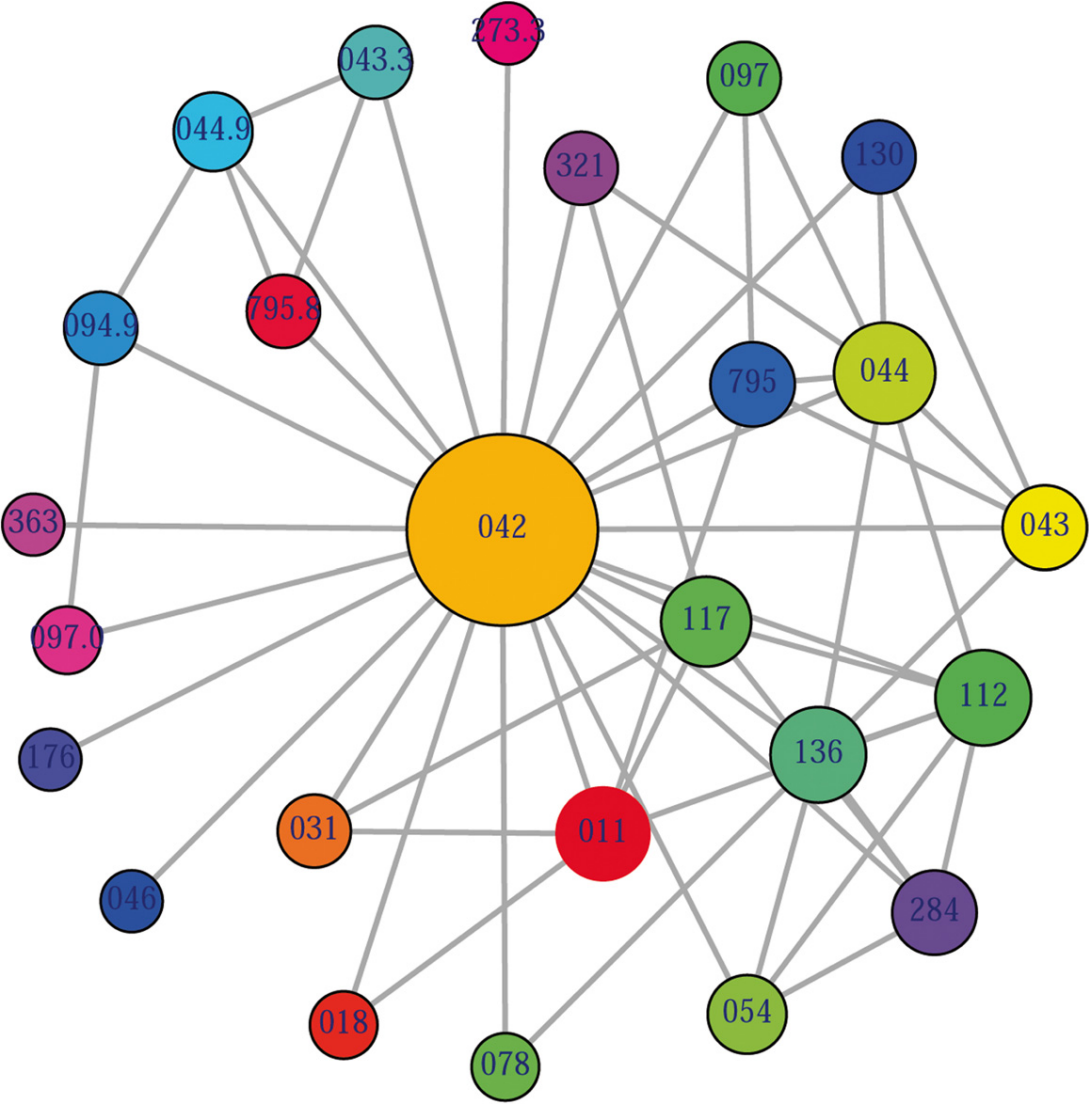
**Output figure of** > comorbidityPatients("042" , "ICD9"). The icd-9-CM code of the HIV is 042, which is used as input to the comorbidityPatients. We show disease comorbidity for the HIV infection.

Patient medical records contain important clarification regarding the co-occurrences of diseases affecting the same patient. Two diseases are connected if they are co-expressed in a significant number of patients in a population [4]. To estimate the correlation starting from disease co-occurrence, we need to quantify the strength of the comorbidity risk. We used two comorbidity measures to quantify the strength of comorbidity associations between two diseases: (i) the Relative Risk (fraction between the number of patients diagnosed with both diseases and random expectation based on disease prevalence) as the quantified measures of comorbidity tendency of two disease pairs; and (ii) *ϕ*-correlation (Pearsons correlation for binary variables) to measure the robustness of the comorbidity association. We used the relative risk *RR*_*i* *j*_ and *ϕ*-correlation *ϕ*_*i* *j*_ of observing a pair of diseases *i* and *j* affecting the same patient. The *RR*_*i* *j*_ allows us to quantify the co-occurrence of disease pairs compared with the random expectation. When two diseases co-occur more frequently than expected by chance, we will get *RR*_*i* *j*_>1 and *ϕ*_*i* *j*_>0. The two comorbidity measures are not completely independent of each other. We included edges between disease pairs for which the co-occurrence is significantly greater than the random expectation based on population prevalence of the diseases. Clinical information is from the http://www.icd9data.com in the ICD-9-CM format and collected from [4]. The function comorbidityPatients of the comoR package is able to take input an OMIM id/3 or 5 digit ICD-9-CM code of a disease or a list of gene symbols/Entrez ids and provides comorbidity pattern of diseases based on the relative risk and *ϕ*-correlation between two diseases. comorbidityPatients requires two parameters id list and id type (see details in the Additional file 1). An example and its output (Figure 2) is as follows: 

### Gene–disease association

**Figure 3:**
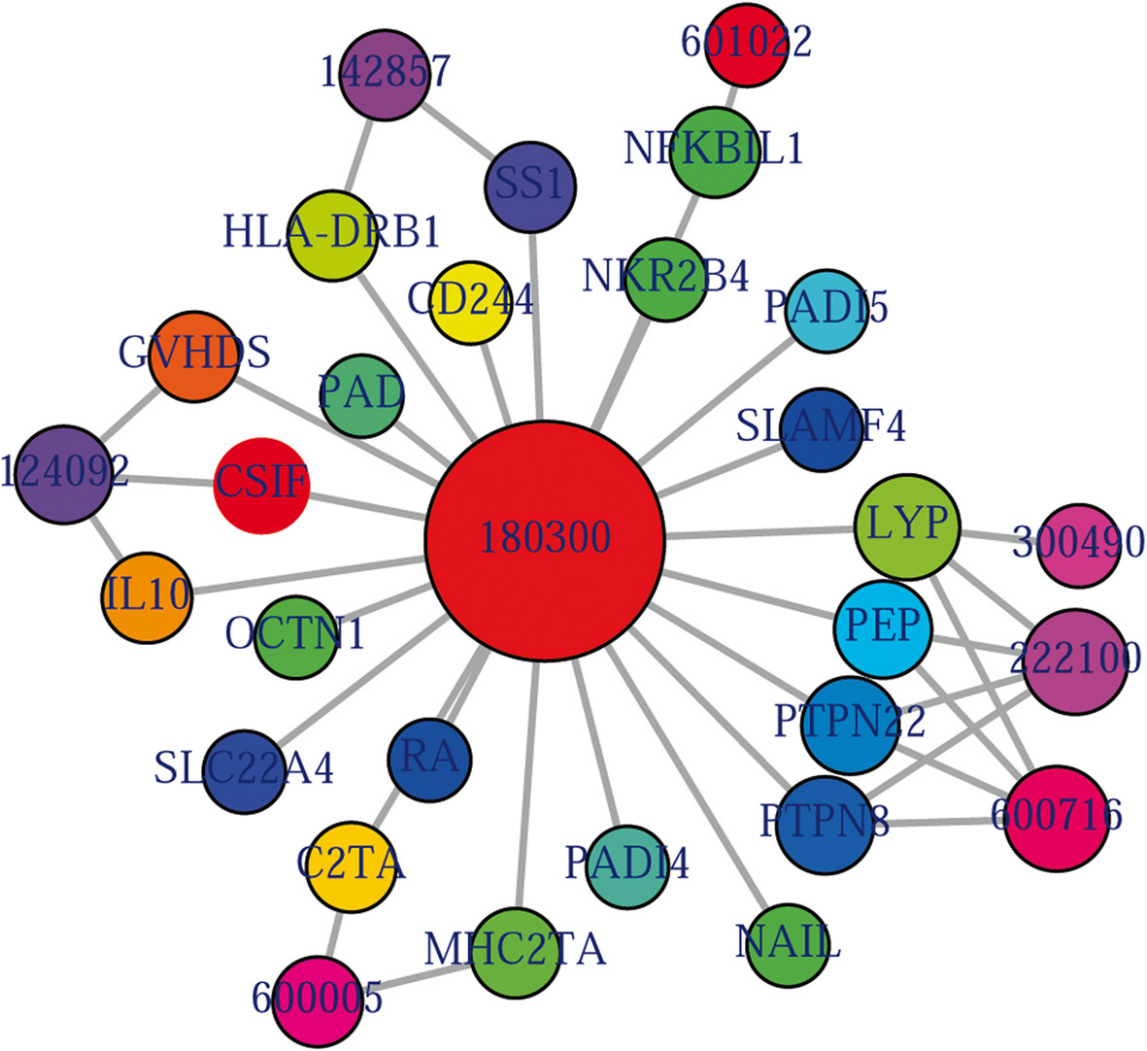
**Output figure of**> comorbidityOMIM("180300" , "OMIM")**.** The OMIM disease id of the Rheumatoid arthritis is 180300, which is used as input to the comorbidityOMIM. We show disease comorbidity for the Rheumatoid arthritis through the gene disease associations.

comoR makes use of OMIM [27] to explore the genetic association between diseases. Two diseases are connected if they share at least one gene that is statistically significant dysregulated [28]. comoR computes disease-disease association by adopting semantic similarity measures and hypergeometric test. OMIM diseases ids are mapped with ICD-9-CM codes based on the literature [3]. Neighbourhood based benchmark method is used to identify the comorbidity pattern among diseases [28]. We build the associated network as a bipartite graph; each common neighbour node is selected based on the Jaccard coefficient method [28]. comorOMIM function of comor takes as input any of these three options: a list of gene symbols, a list of Entrez gene ids or an OMIM id. This function provides disease comorbidity associations and network based on the disease-gene associations. comorOMIM requires two parameters id list and id type (see details in the Additional file 1). An example and its output (Figure 3) is as follows:

### Pathway–disease association

**Figure 4:**
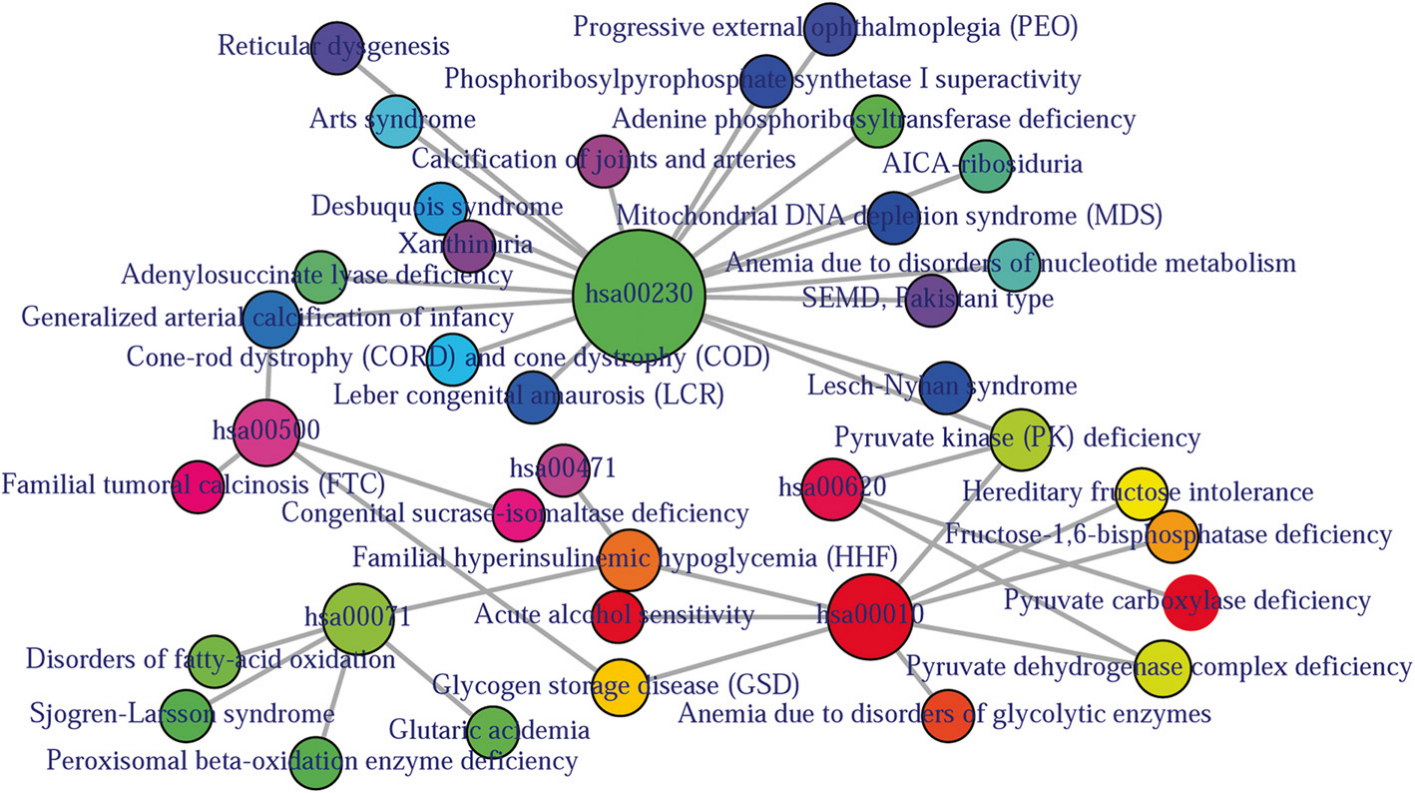
**Output figure of**>comorbidityPath("00010", "Pathway")**.** The kegg pathway id 00010 is used as input to the comorbidityPath. We show disease comorbidity for the pathway "00010" through the pathway disease associations.

The analysis of pathway-disease associations is important to investigate the molecular mechanism of a disease. We have used Kegg pathway and disease database (http://www.genome.jp/kegg/) and developed a function comorbidityPath to predict the comorbidity risk based on disease pathway association [29]. This software identifies the disease–disease associations using the associations among molecular pathways and their associated diseases. Hypergeometric test is used for extracting associations among pathways and diseases; graph topological structure is used to measure the similarity between diseases [30]. comorbidityPath function takes as input any of the following options: a list of gene symbols, a list of Entrez gene ids or an OMIM id. This function provides disease comorbidity associations and network based on the pathway-disease associations. comorbidityPath requires two parameters id list and id type (see details in the Additional file 1). An example and its output (Figure 4) is as follows: 

### Ontology and causal inference to evaluate comorbidity

**Figure 5:**
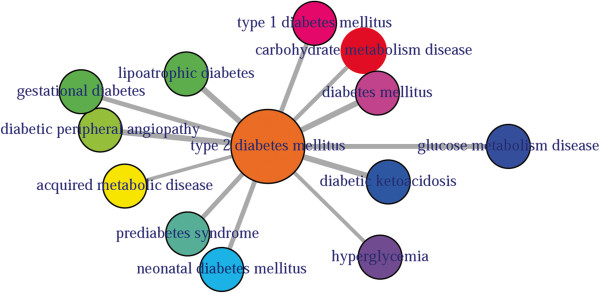
**Output figure of**comorbidityDO("DOID:9352" , "DOID")**.** The DO id of the type 2 diabetes mellitus is DOID:9352, which is used as input to the comorbidityDO. We show disease comorbidity for the type 2 diabetes mellitus using the disease ontology.

**Figure 6:**
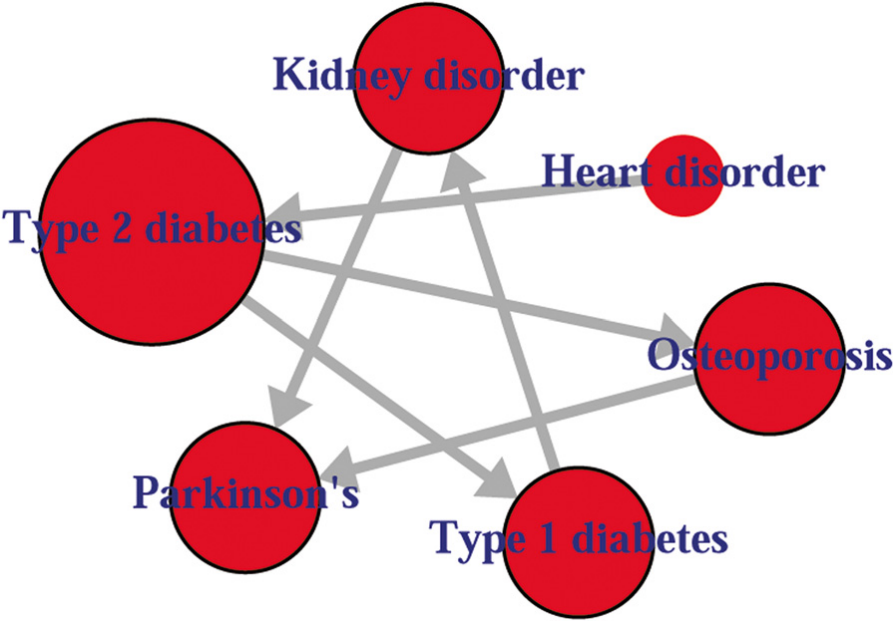
**Output figure of **comorbidityCausality("gmG", "comorbiditydata", "PC")**.** We show cause-effect relationships among 6 diseases.

DO provides an open source ontology for the integration of biomedical data that is associated with human diseases [10]. Terms in DO include disease names and disease-related concepts, which are organised in a directed acyclic graph (DAG) [31]. Disease Ontology Lite (DOLite) gives more interpretable results for gene-disease association tests [32]. DO and DOLite enable us to analyse disease association by adopting semantic similarity measures to expand our understanding of the relationships between different diseases. The semantic comparisons of DO provides quantitative ways to compute similarities between diseases [30]. So we have developed a function comorbidityDO for the computation of DO and DOLite based disease comorbidity in an ontology sense. It is a DO-based enrichment analysis function to measure association among diseases and to explore their functional associations from gene sets. Hypergeometric geometric test is used to compute whether the number of selected genes associated with the DO term is larger than expected. Gene set enrichment analysis are used for predicting the significance of gene–disease and disease–disease associations. comorbidityDO function operates by using either of the following input: DO id, a list of gene symbols or Entrez gene ids of the patient sample. This function provides disease comorbidity associations and network based on the DO and DOLite. comorbidityDO requires two parameters id list and id type (see details in the Additional file 1). An example and its output (Figure 5) is as follows: 

Comorbidity associations among diseases, i.e. the output of comoR, could be a useful input for causal inference software, precisely pcalg to predict the causal inference relationships among the comorbidity diseases. In the comoR, we have included a function comorbidityCausality to predict the causality inference among the diseases using the PC, RFCI, and FCI algorithms of the pcalg [33]. The directed edges of the network show the direction of the cause-effect relationships among diseases. Finally a network disease analysis leads to a patient comorbidity map which is a powerful visualisation of the patient condition. Nodes of the comorbidity map represent diseases and edge between the nodes represents comorbidity risk. Noteworthy, if related molecular information is available, exercise and diet could be also incorporated and be used in the comorbidity map. comorbidityCausality requires two parameters: comorbidity associations of comoR output and preprocessed gene expression data (see details in the Additional file 1). An example and its output (Figure 6) is as follows:

## Methods

We used two comorbidity measures to quantify the strength of comorbidity associations between two diseases - Relative Risk (*RR*_*i* *j*_) as the quantified measures of comorbidity tendency of two disease pairs and *ϕ*-correlation (*ϕ*_*i* *j*_) to measure the robustness of the comorbidity association, which are calculated by using following two equations:

(1) $$RR_{\text{ij}}=\frac{C_{\text{ij}}/N}{(P_{i}P_{j}-C_{\text{ij}})/N^{2}}=\frac{C_{\text{ij}}N}{P_{i}P_{j}-C_{\text{ij}}}$$

(2) $$\phi_{\text{ij}}=\frac{C_{\text{ij}}N-P_{i}P_{j}}{\sqrt{\left(P_{i}P_{j}-C_{\text{ij}})(N-P_{i})(N-P_{j}\right)}}$$

where *N* is the total number of patients in the population, *P*_*i*_ and *P*_*j*_ are incidences/prevalences of diseases *i* and *j* respectively. *C*_*i* *j*_ is the number of patients that have been diagnosed with both diseases *i* and *j*, and *P*_*i*_*P*_*j*_ is the random expectation based on disease prevalence. The significance of the relative risk *RR*_*i* *j*_ is calculated by using the Katz et al. method to estimate confidence intervals [34]. The 99% confidence interval for the *RR*_*i* *j*_ between two diseases *i* and *j* is calculated by: Lower bounds of the confidence interval (*LB*)=*RR*_*i* *j*_∗*exp*(−2.56∗*σ*_*i* *j*_) and Upper bounds of the confidence interval (*UB*)=*RR*_*i* *j*_∗*exp*(2.56∗*σ*_*i* *j*_), where *σ*_*i* *j*_ is given by: $\sigma_{\text{ij}}=\frac{1}{C_{\text{ij}}}+\frac{1}{P_{i}P_{j}}-\frac{1}{N}-\frac{1}{N^{2}}$. Disease pairs within the 99% confidence interval are only considered if the *LB* value is larger than 1 when *RR*_*i* *j*_ is larger than 1, or if the *UB* value is smaller than 1 when *RR*_*i* *j*_ is smaller than 1. For *ϕ*_*i* *j*_>0 comorbidity is larger than expected by chance and for *ϕ*_*i* *j*_<0 comorbidity is smaller than expected by chance. We can determine the significance of *ϕ*≠0 by performing a *t*-test. This consists of calculating *t* according to the formula: $t=\frac{\phi \sqrt{n-2}}{\sqrt{1-\phi^{2}}}$, where *n* is the number of observations used to calculate *ϕ*.

Diseases are connected when the diseases share at least one significant dysregulated gene or signaling pathway. Let a particular set of human diseases *D* and a set of human genes *G*, gene-disease associations attempt to find whether gene *g*∈*G* is associated with disease *d*∈*D*. If *G*_*i*_ and *G*_*j*_, the sets of significant up and down dysregulated genes associated with diseases *i* and *j* respectively, then the number of shared dysregulated genes $\left(n_{\text{ij}}^{g}\right)$ associated with both diseases *i* and *j* is as follows:

(3) $$n_{\text{ij}}^{g}=N(G_{i}\cap G_{j})$$

The co-occurrence refers to the number of shared genes or pathways between two diseases. Each common neighbour is calculated based on the Jaccard Index method to measure the strength of co-occurrence, where association score for a node pair is as:

(4) $$\text{As}s_{i,j}=\frac{N(G_{i}\cap G_{j})}{N(G_{i}\cup G_{j})}$$

Hypergeometric test is implemented for enrichment analysis [31]. It is used to assess whether the number of selected genes or pathways associated with disease is larger than expected. To determine whether any disease annotate a specified list of genes at frequency greater than that would be expected by chance, comoR calculates a p-value using the hypergeometric distribution. Significance of the enrichment analysis is assessed by the hypergeometric test and the *p*−*value* is adjusted by false discovery rate (FDR). The hypergeometric p-value is calculated using the following formula:

(5) p−value=1−∑i=0k−1MiN−Mn−iNn

where *N* is the total number of reference genes, *M* is the number of genes that are associated to the disease of interest, *n* is the size of the list of genes of interest and *k* is the number of genes within that list which are associated to the disease.

Graph-based methods using the topology of DO graph structure is used to compute semantic similarity. We have adapted the method for measuring the functional similarity of protein-coding genes based on GO terms [30]. Semantic values of DO term or diseases were calculated based on the DAG of corresponding diseases. Semantic similarity for any pair of DO term or diseases between *DA* and *DB* is calculated based on disease semantic value. Formally, a DO term or a disease *A* can be represented as a graph *DAG*_*A*_=(*A*,*T*_*A*_,*E*_*A*_), where *T*_*A*_ is the set of all diseases or DO terms in *DAG*_*A*_, including term *A* itself and all of its ancestor terms in the DO graph, and *E*_*A*_ is the set of corresponding edges that connect the DO terms in *DAG*_*A*_. To encode the semantic of a DO term in a measurable format to enable a quantitative comparison, Wang firstly defined the semantic value of term *A* as the aggregate contribution of all terms in *DAG*_*A*_ to the semantics of term *A*, terms closer to term *A* in *DAG*_*A*_ contribute more to its semantics [30]. Thus, we defined the contribution of a disease or DO term *t* in *DAG*_*A*_ to the semantics of DO term *A* as the *D* value of disease or term *t* related to disease or term *A*, *D*_*A*_(*t*), which can be calculated as:

(6) $$\;\left\{\begin{array}{l} D_{A}(A)=1\\ D_{A}(t)=\text{max}\{w_{e}*D_{A}(t^{\prime })|t^{\prime }\in \;\text{children}\;\text{of}(t)\}\;\text{if}\;t\neq A \end{array}\right)$$

where *w*_*e*_ is the semantic contribution factor for edge *e* (*e*∈*E*_*A*_) linking term or disease *t* with its child term or disease $t^{{}^{\prime }}$. It is assigned between 0 and 1 according to the types of associations. Term *A* contributes to its own is defined as one. Then the semantic value of DO term or disease *A*, *DV*(*A*) is calculated as:

(7) $$\text{DV}(A)=\underset{t\in T_{A}}{\sum }D_{A}(t)$$

Thus given two DO terms or diseases *A* and *B*, the semantic similarity between these two terms or disease is defined as:

(8) $$S_{\text{sim}}(A,B)=\underset{t\in T_{A}\cap T_{B}}{\sum }\frac{D_{A}(t)+D_{B}(t)}{\text{DV}(A)+\text{DV}(B)}$$

where *D*_*A*_(*t*) is the semantic value of disease *t* related to DO term or disease *A* and *D*_*B*_(*t*) is the semantic value of DO term or disease *t* associated to DO term or disease *B*.

## Comparison with similar software

An R package “comorbidities” that has functions to categorize comorbidites into the Deyo-Charlson index, the original Elixhauser index of 30 comorbidities, and the AHRQ comorbidity index of 29 diagnoses [35,36]. This package provides total comorbidity count or the total Charlson score. But comoR provides relative risk, *ϕ*-correlation, associated genes, pathway and p-value between the comorbidity diseases. It could provide comorbidity associations among all diseases. So comoR is more useful than “comorbidities”.

Most of the researchers have done the survival analysis and developed tools considering a single infection or disease. Cho et al. developed robust likelihood-based survival modeling for microarray data [18] and Zhang et al. developed Net-Cox model by integrating network information into the Cox’s proportional hazard model for the survival prediction [37]. However, these approaches for analysing the death and recurrence outcomes are based on the single disease (e.g. ovarian cancer). But the survival of a patient depends on the disease comorbidity, treatment plan and environmental effect [38]. To observe the association among diseases through the biomarker genes, we have compared the significance of genes for each disease using network-based Cox regression approach. We have calculated network (genes co-expression and functional linkage networks) based penalised regression coefficient (*β*) values of 5 genes in five diseases conditions(breast cancer, colon cancer, ovarian cancer, liver cancer and osteosarcoma) by using Net-Cox. For this comparative study we have considered five NCBI GEO data sets, accession numbers are GSE3494, GSE17536, GSE26712, GSE10141 and GSE21257 [39-43]. The comparative coefficient (*β*) values of five significant genes (BRCA1, BRCA2, PTEN, TGFB2 and TP53) in 5 diseases conditions are reported in the Table 1. It is observed that diseases may coexist in the same patient. Our software is able to predict occurrence of other diseases in relation to primary disease. So the comorbidity output of our software could be helpful for more accurate survival analysis. So, comoR could be integrate as a pipeline with the survival analysis softwares.

**Table 1:** **Comparative values of genes co-expression and functional linkage network based penalised Cox regression coefficient (*β*) of five significant genes (BRCA1, BRCA2, PTEN, TGFB2 and TP53) in five diseases conditions (breast cancer, colon cancer, ovarian cancer, liver cancer and osteosarcoma)**

**Disease name**	**Network type**	**BRCA1**	**BRCA2**	**PTEN**	**TGFB2**	**TP53**
	Co-expression	8.1253	58.4088	9.9136	31.5791	17.6486
Breast cancer	Functional linkage	1.3637	42.1227	53.2586	19.9091	23.4185
	Co-expression	22.4097	18.3406	17.8181	28.2778	24.0951
Colon cancer	Functional linkage	40.4169	23.6457	37.3934	17.9620	20.2739
	Co-expression	42.5902	155.2418	-0.0751	-0.4850	27.1997
Ovarian cancer	Functional linkage	24.1814	14.8738	33.2762	27.0234	-22.8965
	Co-expression	5.7010	10.2188	41.2701	29.6339	3.2189
Liver cancer	Functional linkage	13.3196	11.4365	7.3683	3.1508	1.9305
	Co-expression	11.8679	10.5565	-1.3561	-8.1221	4.4491
Osteosarcoma	Functional linkage	51.3299	17.1618	15.1504	4.2642	5.3983

## Discussion

Exploring associations among diseases at the molecular and clinical levels could greatly facilitate our understanding of pathogenesis, and eventually lead to better diagnosis and treatment. If two diseases have associated comorbidity, the occurrence of one of them in a patient may increase the likelihood of developing the other diseases. Development of methods integrating genetic and clinical data will assist clinical decision making and represent a large step towards individualised medicine. Hidalgo et al. analysed comorbidity associations using the medical records [4]. To our knowledge, there is no available R software package for the prediction of disease comorbidities. An R package “comorbodoties” is able to categorises ICD-9-CM codes based on published 30 comorbidity indices using Deyo adaptation of Charlson index and the Elixhauser index [35,36]. We have developed comoR, an R package that implements different statistical approach for the prediction of disease comorbidity using divers set of data.

Advances in high-throughput molecular assay technologies in the fields of genomics, proteomics and other omics is increasing the diagnostic and therapeutic strategies, and systems-driven strategies for personalised treatment. In particular, the availability of these data sets for many different diseases presents a ripe opportunity to use data-driven approaches to advance our current knowledge of disease relationships in a systematic way. Patient’s genetic/genomic data is becoming important for clinical decision making, including disease risk assessment, disease diagnosis and subtyping, drug therapy and dose selection [44]. In the future, clinicians will have to consider genetic/genomic implications to patient care throughout their clinical workflow, including electronic prescribing of medications. The identified disease patterns can then be further investigated with regards to their diagnostic utility or help in the prediction of novel therapeutic targets. Therefore, comoR could be helpful for the personalised medicine system. This software will provide us to detect many diseases at the earliest detectable phase, weeks, months, and maybe years before symptoms appear. Thus it could be applicable in the personalised medicine and in clinical bioinformatics.

## Conclusion

Doctors need to be kept updated on novel information on likely comorbidities of diseases. The comoR software provides a robust approach to study disease comorbidities, which can be easily integrated into pipelines for high-throughput and clinical data analysis and to predict causal inference of a disease. This software will help to gain a better understanding of the complex pathogenesis of disease risk phenotypes and the heterogeneity of disease comorbidity. Thus it could be applicable in the personalised medicine and in clinical bioinformatics.

## Availability and requirements

The software package comoR has been written in the platform independent R programming language. It requires R version 3.0.1 or newer to run. The software is freely available at http://www.cl.cam.ac.uk/~mam211/comoR/ and will appear in Comprehensive R Archive Network (CRAN) at (http://cran.r-project.org/).

## Competing interests

The authors declare that they have no competing interests.

## Authors’ contributions

The software was developed by MAM under the supervision of PL. MAM and PL wrote the manuscript. All authors contributed to and approved the manuscript.

## Supplementary Material

Additional file 1 comoRdocumentation. Click here for file
