# Supplementary material for: comoR: a software for disease comorbidity risk assessment
Source: J Clin Bioinforma. 2014 May 23;4:8. doi: 10.1186/2043-9113-4-8 (PMC4081507; doi:10.1186/2043-9113-4-8)
Supplement: Additional file 1 — comoRdocumentation. [file 2043-9113-4-8-S1.pdf]

comoR software documentation

December 16, 2013

`comoR` *comoR software*

Description

This software is able to predict disease comorbidity risk. It also provides pipeline with different causal inference packages to predict the causal relationships among diseases.

Details

Software:	comoR
Type:	Software
Version:	1.1
Date:	2013-12-16
License:	GPL-2

Author(s)

Mohammad Ali Moni and Pietro Lio' <pl219@cam.ac.uk>

Maintainer: Mohammad Ali Moni <mam211@cam.ac.uk> and Pietro Lio' <pl219@cam.ac.uk>

`comorbidityPatient` *Comorbidity between diseases based on the clinical information*

Description

Given a list of OMIM id/3 or 5 digit ICD-9-CM code, returns comorbidity risk scores (relative risk, phi-correlation etc) and comorbidity association network among diseases.

Usage

```
comorbidityPatient(disease id/code, id type )
```

Arguments

`disease id/code`
: one disease OMIM id / 3 or 5 digit ICD-9-CM code

`id type`
: input id type (OMIM / ICD9)

Details

This function calculates relative risk and phi-correlation between diseases.

Value

Comorbidity risk scores (relative risk, phi-correlation etc) and comorbidity association network among diseases.

Author(s)

Mohammad Ali Moni <mam211@cam.ac.uk>, Pietro Lio' <pl219@cam.ac.uk>

References

[1] Hidalgo, Cesar A and Blumm, Nicholas and Barabasi, Albert-Laszlo and Christakis, Nicholas A, Nicholas A, A dynamic network approach for the study of human phenotypes. PLoS computational biology 2009, 5(4), e1000353.

[2] Park, Juyong and Lee, Deok-Sun and Christakis, Nicholas A and Barabasi, Albert-Laszlo, The impact of cellular networks on disease comorbidity. Molecular systems biology 2009, 5(1).

[3] Katz, D., Baptista, J., Azen, S., and Pike, M. (1978). Obtaining confidence intervals for the risk ratio in cohort studies. Biometrics, pages 469-474.

Examples

```
comorbidityPatient("101900","OMIM")

comorbidityPatient("042","ICD9")
```

---

comorbidityOMIM — *Comorbidity between diseases based on the gene-disease associations*

Description

Given a list of gene symbol, Entrez gene id or disease ontology id, returns comorbidity risk and association network among diseases.

Usage

```
comorbidityOMIM(id list, id type)
```

Arguments

`id list`	a list of gene symbols / Entrez id or a disease OMIM id
`id type`	input id type (Symbol / Entrez / OMIM)

Details

This function predicts disease-disease association by adopting semantic similarity measures and hypergeometric test based on the gene-disease association.

Value

Comorbidity risk scores and comorbidity association network among diseases.

Author(s)

Mohammad Ali Moni <mam211@cam.ac.uk>, Pietro Lio' <pl219@cam.ac.uk>

References

[1] McKusick, V. A., Mendelian inheritance in man and its online version, omim. American journal of human genetics 2007, 80(4), 588.

[2] Goh, K.-I., Cusick, M. E., Valle, D., Childs, B., Vidal, M., and Barabasi, Albert-Laszlo, The human disease network. Proceedings of the National Academy of Sciences 2007, 104(21), 8685-8690.

[3] Park, Juyong and Lee, Deok-Sun and Christakis, Nicholas A and Barabasi, Albert-Laszlo, The impact of cellular networks on disease comorbidity. Molecular systems biology 2009, 5(1).

Examples

```
comorbidityOMIM("101900","OMIM")

listofgeneSymbols<-c("TNFSF11", "TNFRSF11B","TNFRSF11A","TGFB1","A2M", "AACP")
comorbidityOMIM(listofgeneSymbols,"Symbol")

listofgeneEntezId<-c("2178", "2200","2242","2260")
comorbidityOMIM(listofgeneEntezId,"Entrez")
```

`comorbidityPath` *Comorbidity between diseases based on the pathway-disease association*

Description

Given a list of gene symbol, Entrez gene id or KEGG pathway id, returns comorbidity risk and association network among diseases.

Usage

```
comorbidityPath(id list, id type)
```

Arguments

`id list`	a list of gene symbols / Entrez id or KEGG pathay id
`id type`	input id type (Symbol / Entrez / Pathway)

Details

This function predicts disease-disease association by adopting semantic similarity measures and hypergeometric test based on the pathway-disease association.

Value

Comorbidity risk scores and comorbidity association network among diseases.

Author(s)

Mohammad Ali Moni <mam211@cam.ac.uk>, Pietro Lio' <pl219@cam.ac.uk>

References

[1] Kanehisa, M., Goto, S., Furumichi, M., Tanabe, M., and Hirakawa, M., Kegg for representation and analysis of molecular networks involving diseases and drugs. Nucleic acids research 2010, 38(suppl 1), D355-D360.

[2] Wang, J. Z., Du, Z., Payattakool, R., Philip, S. Y., and Chen, C.-F. (2007). A new method to measure the semantic similarity of go terms. Bioinformatics, 23(10), 1274-1281.

Examples

```
listofPathways<-c("00030", "00051","00230","00270")
comorbidityPath("101900","Pathway")

listofgeneSymbols<-c("TNFSF11", "TNFRSF11B","TNFRSF11A","TGFB1","A2M", "AACP")
ccomorbidityPath(listofgeneSymbols,"Symbol")

listofgeneEntezId<-c("2178", "2200","2242","2260")
comorbidityPath(listofgeneEntezId,"Entrez")
```

`comorbidityDO` *Comorbidity between diseases based on the DO and DOLite*

Description

Given a list of gene symbol, Entrez gene id or disease ontology id, returns comorbidity risk and association network among diseases.

Usage

```
comorbidityOMIM(id list, id type)
```

Arguments

`id list`	a list of gene symbols / Entrez id or a DO id
`id type`	input id type (Symbol / Entrez / DO)

Details

This function predicts disease-disease association by adopting semantic similarity measures and hypergeometric test based on the DO and DOLite.

Value

Comorbidity risk scores and comorbidity association network among diseases.

Author(s)

Mohammad Ali Moni <mam211@cam.ac.uk>, Pietro Lio' <pl219@cam.ac.uk>

References

[1] Du, P., Feng, G., Flatow, J., Song, J., Holko, M., Kibbe, W. A., and Lin, S. M., From disease ontology to disease-ontology lite: statistical methods to adapt a general-purpose ontology for the test of gene-ontology associations. Bioinformatics 2009, 25(12), i63-i68.

[2] Schriml, L. M., Arze, C., Nadendla, S., Chang, Y.-W.W., Mazaitis, M., Felix, V., Feng, G., and Kibbe, W. A., Disease ontology: a backbone for disease semantic integration. Nucleic acids research 2012, 40(D1), D940-D946.

[3] Wang, J. Z., Du, Z., Payattakool, R., Philip, S. Y., and Chen, C.-F., A new method to measure the semantic similarity of go terms. Bioinformatics 2007, 23(10), 1274-1281.

Examples

```
comorbidityDO("DOID:1474","DO")

listofgeneSymbols<-c("TNFSF11", "TNFRSF11B","TNFRSF11A","TGFB1","A2M", "AACP")
comorbidityDO(listofgeneSymbols,"Symbol")

listofgeneEntezId<-c("2178", "2200","2242","2260")
comorbidityDO(listofgeneEntezId,"Entrez")
```

`comorbidityCausality` *Causal inference relationship among diseases*

Description

Given gene expression data and comorbidity output of the comoR software, predicts causal inference relationship among diseases.

Usage

```
comorbidityCausality(gene.data, comordata, method)
```

Arguments

`gene.data`	gene expression data of disease condition
`comordata`	Output of any one function (comorbidityPatient / comorbidityOMIM / comorbidityPath / comorbidityDO) of the comoR
`method`	method type (PC, FCI or RFCI algorithm)

Details

This function predicts causal inference relationship among the diseases using the result of comoR functions and observed gene expression data.

Value

Causal relationship network among diseases.

Author(s)

Mohammad Ali Moni <mam211@cam.ac.uk>, Pietro Lio' <pl219@cam.ac.uk>

References

[1] Kalisch, M., Machler, M., Colombo, D., Maathuis, M. H., and Buhlmann, P., Causal inference using graphical models with the r package pcalg. Journal of Statistical Software 2012, 47(11), 1-26.

See Also

comorbidityPatient, conorbidityOMIM, comorbidityPath, comorbidityDO

Examples

```
library("pcalg")
data("gmG")
comorbiditydata<-comorbidityOMIM("101900","OMIM")
comorbidityCausality("gmG","comorbiditydata","PC")
```

Index

∗Topic **comoR**
comoR, 1
comorbidityCausality, 6
comorbidityDO, 5
comorbidityOMIM, 2
comorbidityPath, 4
comorbidityPatient, 1

comoR, 1
comorbidityCausality, 6
comorbidityDO, 5
comorbidityOMIM, 2
comorbidityPath, 4
comorbidityPatient, 1
